# A Case of Multiple Myeloma Following Bladder Cancer

**Published:** 2016-04-01

**Authors:** Hamid Shafi, Mohsen Vakili Sadeghi, Hosein Ghorbani, Majid Sharbatdaran

**Affiliations:** 1 Urologist, Associate Professor of Urology, Hazrat Fatemeh Zahra Infertility Research Center, Babol University of Medical Sciences, Babol, Iran; 2 Hematologist and Medical Oncologist, Associate Professor of Medicine, Babol University of Medical Sciences, Babol, Iran; 3 Pathologist, Associate Professor of Pathology, Babol University of Medical Sciences, Babol, Iran; 4 Pathologist, Assistant Professor of Babol University of Medical Sciences, Babol, Iran

**Keywords:** Bladder cancer, Multiple myeloma, Synchronous malignancies

## Abstract

Second primary malignancy following multiple myeloma (MM) was reported several years ago. There are also rare reports of cases with synchronous MM and other malignancies. To our knowledge, only one case of MM following bladder cancer has been reported in the literature. Here, we report the second case occurred three months after diagnosis of bladder cancer.

## Introduction

 Multiple Myeloma (MM) is a malignant hematologic disease due to clonal expansion of bone marrow plasma cells.^1^ Another primary malignancies were reported before,^2^ synchronous ^3^^,^^4^ and following MM diagnosis.^5^^,^^6^ Acute myeloid leukemia and myelodysplastic syndrome following MM treatment were recognized several years ago. Also, other second primary malignancies may occur following MM.^7^^-^^9^ On the other hand, MM following another malignancy is rare and less than 30 cases have been reported on PubMed Medline.^10^ To our knowledge, only one case of MM following bladder cancer^10^ has been reported in the literature and now we report the second case.

**Case presentation**

**Figure 1:**
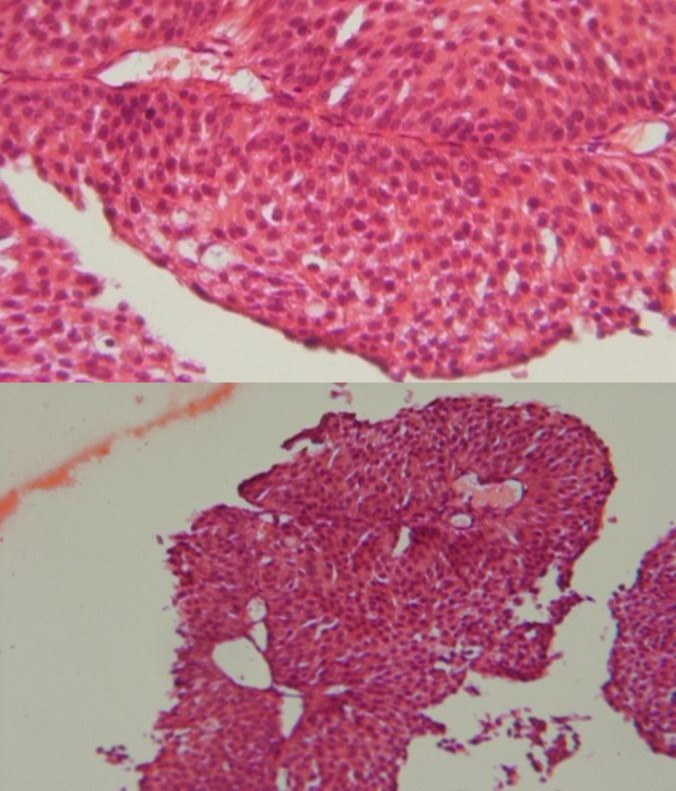
H&E staining, transitional cell carcinoma of bladder

**Figure 2-A:**
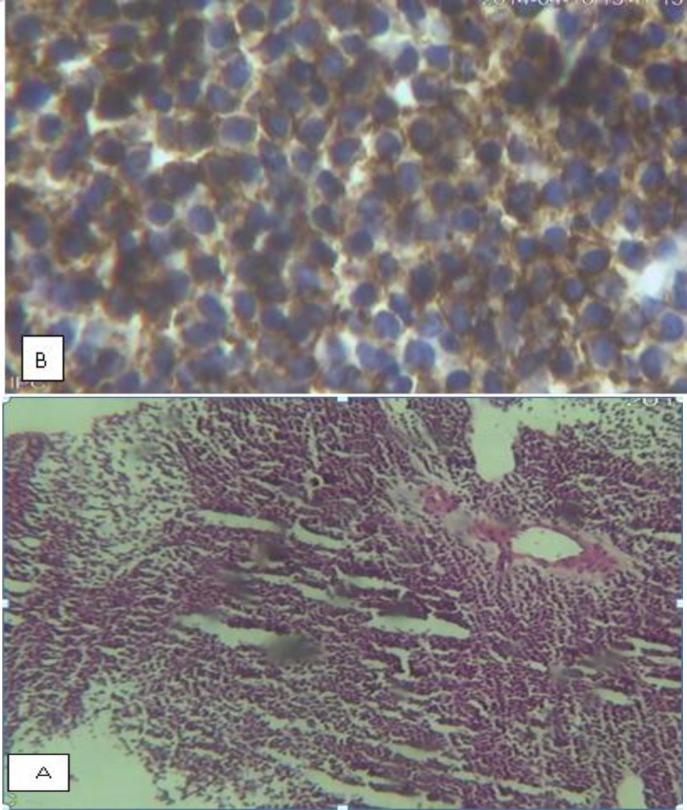
Paravertebral tumor histology (H&E), B: Paravertebral tumor IHC (CD138)

**Figure 3:**
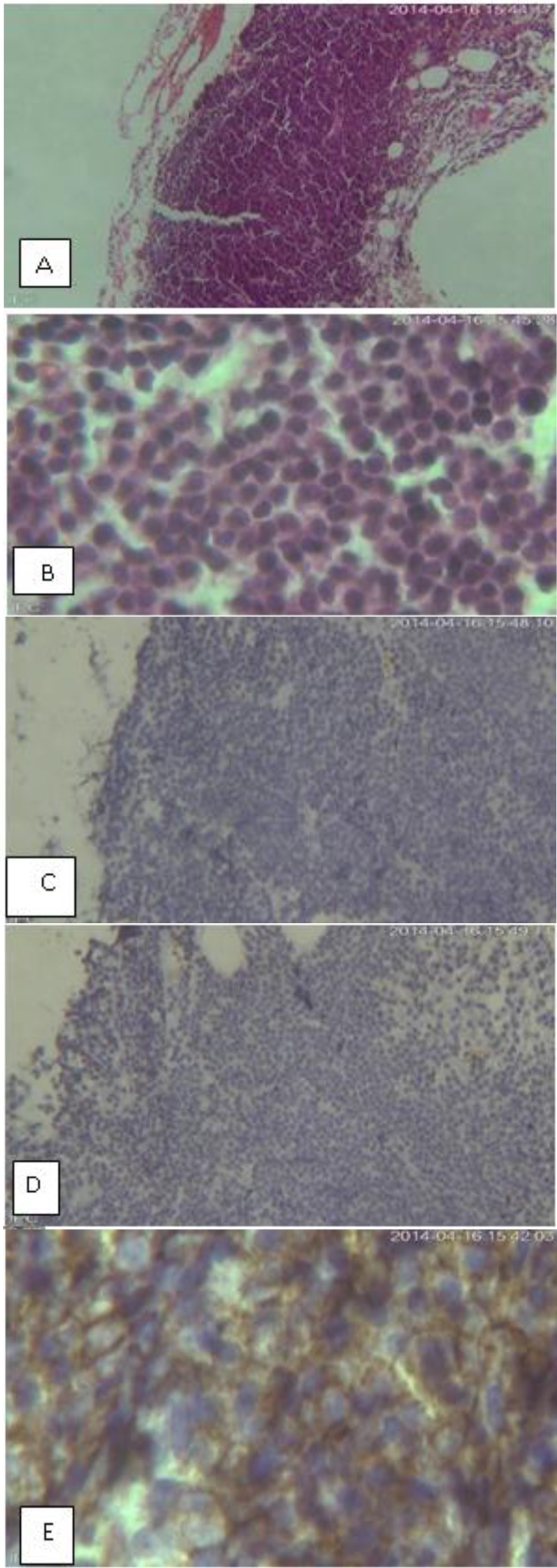
A and B: Bone marrow biopsy C: IHC (CD45), D: IHC (CD20), E: IHC (CD138)

 A 56-year- old woman was admitted to Beheshti general hospital of Babol because of gross hematuria at 92/8/2. Cystoscopy was done and several tumors were seen in floor and lateral wall of the bladder .Transurethral resection of bladder tumor was done and pathologist reported “noninvasive low grade transitional cell carcinoma” (Figure 1). The patient treated with 6 courses of intravesical BCG (190 mg bovis BCG in 50 cc normal saline). Follow-up cystoscopy was done and no residual tumor was seen. Two months after bladder cancer diagnosis, she developed back pain that gradually increased, so that she eventually became bedridden. She lost 8 kilograms of her weight over 2 months and also complained of excessive sweating. Chest CT scan on 22.01.2014 revealed 7th thoracic vertebral destruction and a 5 X 5 cm paraspinal tumor adjacent to destroyed vertebra. Core needle biopsy from paraspinal tumor was done and "small round cell tumor" was reported in pathology. In immunohistochemistry, CD138 was positive and CD45, CD20 and CD3 were negative (Figure 2). Finally, plasmacytoma was diagnosed. Bone marrow biopsy and immunohistochemistry (IHC) were done. Nodular involvement of bone marrow with monomorph lymphocytoid cells was reported and IHC report was the same as paravertebral tumor (Figure 3).

**Figure 4:**
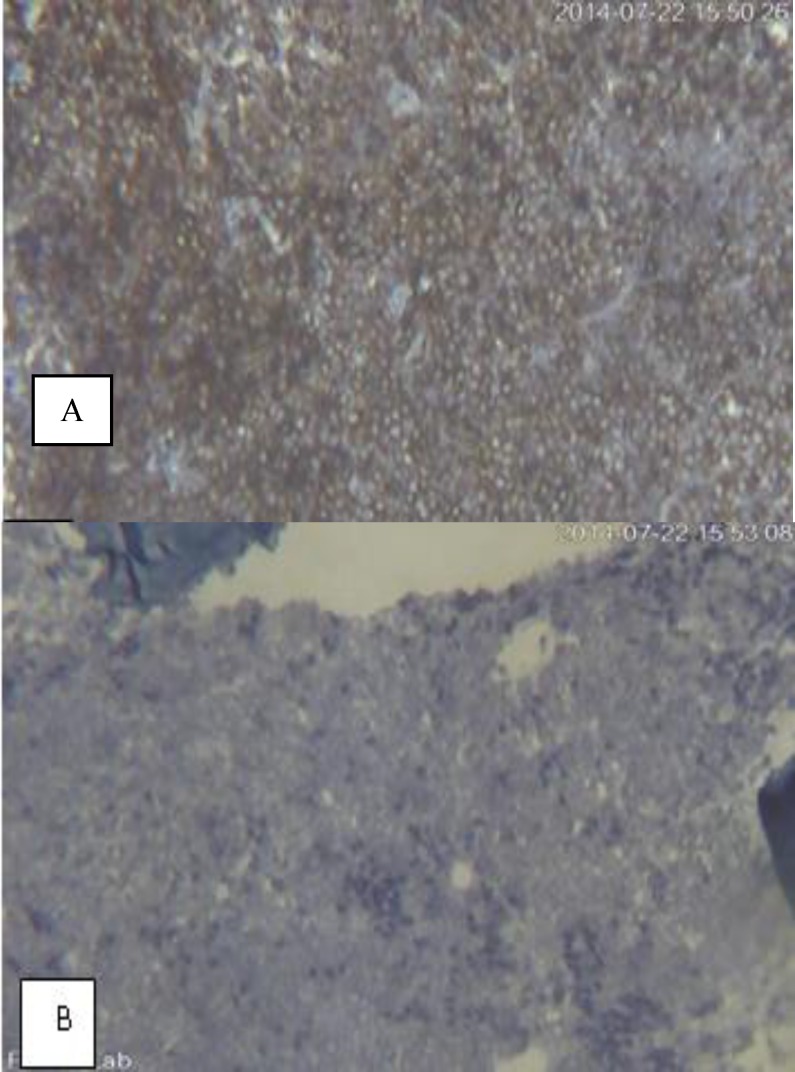
IHC for lambda (A) and kappa (B) light chain

Immunohistochemistry for kappa and lambda light chain revealed only positive lambda chain on bone marrow biopsy and confirmed monoclonality of lymphocytoid cells (Figure 4). Serum and urine protein electrophoresis were normal. Abdominal sonography revealed two hyperechoic masses (50 X 57 mm and 20 X 23 mm) in the liver. Abdominal CT scan with hemangioma protocol indicated changes in favor of hepatic hemangiomas. Other lab tests were:

**Table 1:** laboratory tests of the patient

WBC	7100
Hemoglobin	10.1 gr/dl
MCV	91 fL
Platelet	231000
ESR	29
LDH	380 (in normal range)
Uric acid	10.1 mg/dl
Creatinine	1.4mg/dl
β2 Microglobulin	4.96
Liver function tests	normal
Serum immunoglobulin level	Serum IgG: 4684 mg/dl (normal range: 700-1600), IgA: 40 mg/dl (70-400), serum IgM: 13 mg/dl (40-230)

Because of sever back pain and danger of cord compression, radiotherapy was begun as first treatment and then chemotherapy was done with DT-PACE protocol (dexametason, thalidomide, cisplatin, adriamycin, cyclophosphamid, etoposide). After chemotherapy, the patient was admitted for neutropenic fever. On the 4th day following admission, she suddenly developed loss of consciousness associated with increased blood pressure and miotic pupils (probably brain hemorrhage occurred) and the patient was expired before any imaging from her head.

## Discussion

 Sometimes multiple myeloma occurs prior to, synchronous with, or after solid tumor diagnosis. Agarwal et al. reported one case of “Synchronous Presentation of Multiple Myeloma and Lung Cancer”. In their literature review, they found 5 other cases with synchronous MM and one solid tumor: breast cancer, lung cancer, prostate cancer, colon cancer and squamous cell carcinoma of the pyriform sinus.^3^ Todoli et al. reported 6.2% of second primary malignancy in the 210 MM patients.^8^

MM following solid tumors is extremely rare. Yihao wang et al. reported first case of MM seventeen months after diagnosis of bladder cancer. In a review, they found only 5 case reports of multiple myeloma following solid tumors: a lung cancer, a prostate cancer, a penile myeloid sarcoma, a colon cancer and a gastrointestinal stromal tumor.^10^

Most prior malignancies in 23 patients before MM diagnosis were hematologic cancer including chronic lymphocytic leukemia, myeloproliferative disorders and lymphoma.^10^ when second primary malignancy occurs after MM diagnosis, drug toxicity may be a potential causal factor,^5^^,^^6^ but in other situations, no etiologic correlation between MM and another malignancy has been known. The patient that Mr. Wang reported was treated with local Farmorubicin, and MM was diagnosed 17 months after bladder cancer diagnosis, but our patient was treated with BCG, and MM was diagnosed only after 3 months. This time period is too short that actually we can say, our patient had bladder cancer and multiple myeloma simultaneously. Moreover, we cannot postulate MM as a treatment complication of bladder cancer. One explanation may be a common carcinogen as etiologic factor for MM and another solid tumor.

In our case, serum IgG level was very high but serum protein electrophoresis was negative, which might be due to a laboratory error.

## CONCLUSION

 We report a rare case of multiple myeloma following bladder cancer with a short period of time between two diseases. Simultaneous malignancies may result from same carcinogens. Further studies are needed to identify this idea and probable carcinogens.
